# Detailed statistical analysis plan for the Dutch STRIDER (Sildenafil TheRapy In Dismal prognosis Early-onset fetal growth Restriction) randomised clinical trial on sildenafil versus placebo for pregnant women with severe early onset fetal growth restriction

**DOI:** 10.1186/s13063-018-3136-z

**Published:** 2019-01-11

**Authors:** Anouk Pels, Janus C. Jakobsen, Wessel Ganzevoort, Christiana A. Naaktgeboren, Wes Onland, Aleid G. van Wassenaer-Leemhuis, Christian Gluud

**Affiliations:** 1 Department of Obstetrics and Gynecology, Amsterdam UMC, University of Amsterdam, Amsterdam, The Netherlands; 2 The Copenhagen Trial Unit, Centre for Clinical Intervention Research, Rigshospitalet, Copenhagen University Hospital, Copenhagen, Denmark; 3 Department of Cardiology, Holbæk Hospital, Holbaek, Denmark; 4 Department of Neonatology, Emma Children’s Hospital/Academisch Medisch Centrum, Amsterdam, The Netherlands

**Keywords:** Fetal growth restriction, Placental insufficiency, Sildenafil, Randomised placebo-controlled trial, Statistical analysis plan

## Abstract

**Objective:**

The objective of the Dutch Sildenafil therapy in dismal prognosis early onset fetal growth restriction (STRIDER) randomised clinical trial is to assess the beneficial and harmful effects of sildenafil versus placebo on fetal and neonatal mortality in pregnant women with severe early-onset fetal growth restriction. The objective of this detailed statistical analysis plan is to minimize the risks of selective reporting and data-driven analysis.

**Setting:**

The setting is 10 tertiary care hospitals and one secondary care hospital in The Netherlands.

**Participants:**

The participants will be 360 pregnant women with severe early-onset fetal growth restriction.

**Interventions:**

The intervention is sildenafil 25 mg or placebo orally three times a day.

**Primary and secondary outcome measures:**

The primary outcome is a composite of death or major neonatal morbidity assessed at hospital discharge. The secondary outcomes are neurodevelopmental impairment; mean scores of the Bayley III cognitive and motor assessment; the proportion of patients experiencing either preeclampsia or haemolysis, elevated liver enzymes, and low platelets syndrome; pulsatility index of uterine arteries, umbilical artery, and middle cerebral artery; birthweight; and gestational age at either delivery or intra-uterine death.

**Results:**

A detailed statistical analysis is presented, including pre-defined exploratory outcomes and planned subgroup analyses. One interim analysis after 180 patients had completed the study was planned and a strategy to minimise the risks of type I errors due to repetitive testing is presented. During review of this manuscript the interim analysis was performed by the Data Safety Monitoring Board and early stopping of the trial was recommended. Final analyses will be conducted independently by two statistically qualified persons following the present plan.

**Conclusion:**

This pre-specified statistical analysis plan was written and submitted without knowledge of the unblinded data and updated after stopping of the trial at interim analysis.

**Trial registration:**

ClinicalTrials.gov, NCT02277132. Registered on 29 September 2014.

**Original protocol for the study**: doi:10.5281/zenodo.56148

## Background

The Dutch Sildenafil therapy in dismal prognosis early-onset fetal growth restriction (STRIDER) randomised clinical trial is a blinded trial was recruiting patients recently, assessing the benefits and harms of sildenafil versus placebo in pregnant women with severe early-onset fetal growth restriction (FGR) and their offspring. The primary outcome is mortality and morbidity of the children. Fetal growth restriction is a condition in which a fetus does not reach its designated growth potential and thus is too small for gestational age (SGA), mostly defined as either estimated fetal weight or abdominal circumference determined by ultrasound below the third percentile or gestational age below the tenth percentile. However, no unanimously agreed definition has yet been adopted [1].

The predominant cause of fetal growth restriction, particularly at early onset (< 32 weeks), is placental dysfunction with high resistance, low-flow, placental circulation, due to inadequate spiral artery remodelling early in pregnancy [2]. Depending on the gestational age at development, the fetus has a substantial risk of mortality and morbidity [3]. As the phosphodiesterase 5- (PDE5-) inhibitor sildenafil causes vasodilatation, it might improve the utero-placental circulation in fetal growth restriction resulting in improved growth and increased chances of healthy survival of the fetus [4–20].

A recent meta-analysis of sildenafil in fetal growth restriction has been published [21]. This meta-analysis included only one randomised clinical trial of sildenafil in which a single administration of 50 mg sildenafil versus placebo was given to pregnant women with fetal growth restriction between 24 and 37 weeks of gestation [22]. An improvement of the Doppler measurements of the umbilical artery and middle cerebral artery was seen in the sildenafil group compared with the placebo group [22]. However, no patient-centred or clinically relevant outcomes (such as morbidity and mortality) were assessed and patients only received a single dose of sildenafil. The review, furthermore, described a non-randomised comparative study in which 10 women received sildenafil 25 mg three times a day compared to 17 women without sildenafil administration [23]. This observational study indicated an increase in fetal abdominal circumference growth and a trend toward better survival in the sildenafil group compared to the group that was untreated [23]. The review does not identify other clinical trials of sildenafil in fetal growth restriction and concludes that more randomised clinical trials are needed [21].

Besides the short-term randomised clinical trial and the observational study mentioned above, we identified one recently published clinical trial where 35 patients with fetal growth restriction were randomised to three groups, receiving either oral sildenafil, transdermal nitroglycerin, or oral placebo [24]. The outcomes were non-validated surrogate outcomes [25], i.e. Doppler ultrasound measurements of the uterine arteries, umbilical artery, and middle cerebral artery were evaluated after administration of the trial interventions. Positive effects of sildenafil and nitroglycerin were seen in the pulsatility index of the uterine artery and the umbilical artery, while no effect was seen in the placebo group [24].

A couple of randomised clinical trials on sildenafil have been conducted in women with diagnosed preeclampsia. A randomised clinical trial including 100 women with preeclampsia showed a statistically significant difference in pregnancy prolongation of 4 days in favour of the sildenafil group compared with the placebo group [26]. In another randomised clinical trial, 35 patients with preeclampsia received sildenafil in increasing dose versus placebo. This trial did not find a significant difference in pregnancy prolongation after treatment with sildenafil compared with placebo [12].

Apart from sildenafil, interest has also focused on L-arginine, which is an amino-acid that interacts in the same pathway as sildenafil and theoretically could have a similar clinical effect. The aforementioned meta-analysis of Chen and colleagues included eight randomised clinical trials and one quasi-randomised study (total 576 patients) assessing L-arginine versus placebo or no therapy [21]. The analysis showed that L-arginine seems to have a significant beneficial effect on birthweight, gestational age at delivery, intracranial haemorrhage, and neonatal respiratory distress syndrome [21]. However, the authors of the meta-analysis state that four of the nine studies were of uncertain quality and there is a high risk of bias [27–30]. Furthermore, the number of randomised patients in the trials is relatively small.

By reviewing the existing literature, high-quality evidence is pending for a pharmacological treatment of fetal growth restriction. Apart from the Dutch STRIDER, four other STRIDER trials are presently conducted or are in different phases of preparation, recruitment, and analysis [31]. The results of the UK STRIDER trial have been published recently [32] and did not show a difference in pregnancy prolongation between patients allocated to sildenafil versus placebo. To minimise the risks of selective reporting and data-driven analyses, we will here shortly describe the plans for interim analysis and in detail our statistical analysis plans of the Dutch STRIDER trial and how the results will be reported. At first submission of this manuscript, the Dutch STRIDER trial was still recruiting patients and collecting the data; however, during the review of this manuscript, the trial was stopped early based on advice of the DSMB.

### Trial overview

Please see the published protocol of the trial for a detailed description of the methodology [33]. In short, the Dutch STRIDER trial compares 25 mg sildenafil three times daily orally with matching placebo three times daily in women with severe early-onset fetal growth restriction. The placebo matches the sildenafil in form, size, colour, smell, and solubility. The patients eligible for inclusion are women from 20 weeks and 0 days of gestation until 29 weeks and 6 days, with fetal growth restriction and signs of placental insufficiency, without an alternative explanation for the fetal growth restriction. Participants will use study medication until 32 weeks of gestation or delivery, whichever comes first. The participants, the treatment providers, the outcome assessors, the statisticians, and the conclusion drawers were planned to be blinded for the treatment allocation [27, 28, 34–40]. The treatment allocation was unblinded on early stopping of the trial. The participants, treatment providers, and outcome assessors were blinded up to stopping the trial at the interim analysis.

The original protocol of the Dutch STRIDER trial was approved by the local ethical committee on 22 July 2014. The first patient was included on 20 January 2015. The trial was conducted according to the principles of the Declaration of Helsinki Medical, Dutch legislation on medical research involving human subjects [41–44] and good clinical practice guidelines (GCP) [45]. Patients could only be included in the trial after written informed consent from the pregnant woman was obtained. All study sites are monitored by an independent clinical research associate of the Nederlandse Vereniging voor Obstetrie en Gynaecologie Consortium. An independent data safety monitoring board (DSMB) monitored the study progress, with a special focus on safety (see below). The trial will be reported according to the Consolidated standards of reporting trials (CONSORT) guidelines [46].

### Intervention period and data collection

The intervention is sildenafil 25 mg three times daily orally versus placebo three times daily up to 32 weeks gestation or delivery, whichever comes first. Clinical outcome data will be recorded from mother and neonate until discharge to home. Follow up of the child will be assessed at 2 years of age in an outpatient setting.

### Concomitant treatments

Patients who participate in the Dutch STRIDER trial will furthermore be treated according to local protocol. The caregivers, blinded to the allocated therapy, will make decisions on the administration of corticosteroids for fetal lung maturity at the moment of delivery, based on fetal and maternal condition and maternal treatment of hypertensive disorder, according to the clinical practice in that particular centre, as if patients were not participating in a trial.

### Baseline variables

The baseline criteria that are considered to be relevant and are planned to be reported are listed in Table 1. The baseline characteristics will be presented by treatment allocation. Binary and categorical outcomes will be expressed in frequencies and percentages. In the case of missing data, there will be a note on how many data were available. Continuous variables will be expressed by either mean and standard deviation (normal distribution) or median and IQR (non-normal distribution). Differences in the treatment arms will not be statistically tested.

**Table 1:** Baseline criteria

	Sildenafil (*n* =)	Placebo (*n* =)
Age (years)		
BMI (kg/m^2^)		
Ethnicity		
Caucasian (%)		
African descent (%)		
Asian (%)		
Other (%)		
Highest completed educational level mother		
High (%)		
Middle (%)		
Low (%)		
Unknown (%)		
Highest completed educational level father/partner		
High (%)		
Middle (%)		
Low (%)		
Unknown (%)		
Language spoken at home		
Only Dutch		
Only other language than Dutch		
More than one language, including Dutch		
Maternal smoking (%)		
Gestational age at inclusion (weeks + days)		
Estimated fetal weight at ultrasound (gram)		
Fetal abdominal circumference at ultrasound (mm)		
Notching uterine artery (one-or two-sided) (%)		
PI umbilical artery > 95th centile (%)		
PI middle cerebral artery < 5th centile (%)		
End-diastolic flow		
Positive (%)		
Absent (%)		
Reversed (%)		
Pregnancy-induced hypertension (%)		
Preeclampsia (%)		
HELLP syndrome (%)		
Systolic blood pressure (mmHg)		
Diastolic blood pressure (mmHg)		

*BMI* body mass index, *PI* pulsatility index, *HELLP* haemolysis, elevated liver enzymes, and low platelets syndrome

### Data collection and storage

Data management was implemented according to GCP guidelines. Patient data up to hospital discharge and long-term follow up data are entered via an electronic case record form (CRF) in a central GCP-proof web-based database to facilitate on-site data entry (RedCap). Security is guaranteed with login names, login codes, and encrypted data transfer. Data collection is performed at multiple time points: at the time of inclusion and randomisation, during the study medication treatment period, at hospital discharge of the child, and at 2 years of corrected age for follow up. Data on eligible patients not included in the study are also recorded, including patient characteristics and the primary outcome (death or survival with major morbidities).

Serum placental growth factor (PlGf) will be analysed after completion of the study. The PlGf analysis currently is not part of standard care and is not often performed. To investigate the predictive value of PlGf for adverse outcomes in FGR, blood serum samples at inclusion are collected and stored. Samples will not be used before the inclusion of participants in the study and data collection is complete.

### Primary outcome

The primary outcome is a composite outcome consisting of either:

1. Neonatal mortality assessed at the time point when the neonate is discharged from the hospital or
2. Major neonatal morbidity defined as
  Intraventricular haemorrhage (IVH) grade 3 or more or
  Periventricular leukomalacia (PVL) grade 2 or more or
  Moderate or severe bronchopulmonary dysplasia (BPD) or
  Necrotising enterocolitis (NEC) grade 2 or more or
  Retinopathy of prematurity (ROP) treated by surgery or laser therapy

- Intraventricular haemorrhage (IVH) and periventricular leukomalacia (PVL) will be assessed in neonates were born at a gestational age < 32 weeks or with birth weight < 1500 g. These neonates will have an ultrasound scan of the brain as standard. Brain magnetic resonance imaging (MRI) will be performed in case different types of abnormalities are seen on ultrasound or in the clinical behaviour of the neonate. The timing and the number of investigations is dependent on the gestational age at birth, the abnormalities seen, and the clinical behaviour of the neonate. Investigations will be performed according to Dutch national recommendations [47]. If a neonate is evaluated by ultrasound, the scan showing the most severe abnormalities will be used to assess neurological morbidity. If a neonate does not have an ultrasound scan because it is born (near-)term and there is no clinical suspicion of neurological morbidity, this will be diagnosed as “no neurological morbidity”.
- Bronchopulmonary dysplasia is assessed at 36 weeks postmenstrual age (PMA) according to the Dutch guideline for BPD and the National Institute of Child Health and Human Development (NICHD) consensus statement using the classification of severity and, if indicated, the oxygen reduction test as described by Walsh et al. [48–53]. Neonates that will be born after 36 weeks gestational age will be diagnosed as “no bronchopulmonary dysplasia”.
- Retinopathy of prematurity (ROP) screening will take place according to the Dutch guideline for ROP [54]. Screening will be performed by an ophthalmologist in neonates born < 30 weeks gestational age and/or with birthweight < 1250 g. Neonates born between 30 and 32 weeks and with birthweight between 1250 and 1500 g will in some situations be screened for retinopathy of prematurity as well. The timing and number of assessments is dependent on the gestational age at birth and the abnormalities found at assessment. Neonates that will not be screened for ROP according to the guideline, will be diagnosed as “no retinopathy of prematurity”.
- Necrotising enterocolitis is a clinical diagnosis and staging will be according to the Bell system [55]. Whether a neonate will have had an episode of necrotising enterocolitis requiring surgery will be assessed and reported at the time of discharge from the neonatal intensive care.

### Secondary outcomes

The secondary outcomes are:

1. The proportion of neonates with neurodevelopmental impairment at 2 years of age, assessed on the two-year Bayley scales of infant development (BSID)-III [56]. Neurodevelopmental follow up will be at the outpatient clinic at the corrected age of the infant of 2 years (2 years after the term age), which is standard in The Netherlands for children born < 30 weeks gestation or born with weight < 1000 g. Neurodevelopmental impairment will be defined using two measures: first, as a cognitive Bayley III score < 85 (or an estimated cognitive delay of more than 3 months when a Bayley test cannot be carried out), composite motor score < 85, cerebral palsy, with a Gross Motor Function Classification System (GMFCS) grade > 1, hearing loss needing hearing aids, or severe visual loss (legally certifiable as blind or partially sighted). The second definition of NDI is similar except it does not include the motor score < 85. Second, we will describe the different components of the composite outcome, including all cases of CP and their GMFCS classifications.
2. The mean composite cognitive Bayley III score (continuous outcome), assessed at the 2-year Bayley scales of infant development BSID-III [56].
3. The mean composite motor score for the Bayley scales of infant development BSID-III [56], and the mean standard scores on the fine and gross motor subscales.
4. The proportion of mothers experiencing either preeclampsia or haemolysis, elevated liver enzymes, and low platelets (HELLP) syndrome. Preeclampsia is defined as hypertension in combination with proteinuria. Hypertension is defined as systolic blood pressure > 140 mmHg and/or diastolic blood pressure > 90 mmHg (Korotkoff V), measured at least twice, after 20 weeks of gestation in a patient that had no hypertension before. Proteinuria is defined as ≥ 300 mg protein measured on 24-h urine collection [57]. HELLP syndrome is defined as elevated lactate dehydrogenase (LDH); either elevated aspartate aminotransferase (AST) or alanine aminotransferase (ALT); and low platelets, according to local laboratory reference values [58]. Second, the proportion of patients with preeclampsia and the proportion of patients with HELLP syndrome will be reported individually as well.
  Whether or not a patient will have had preeclampsia or HELLP syndrome will be assessed when the mother is discharged to go home after delivery. Development of preeclampsia or HELLP syndrome after discharge home for which readmission is necessary will be considered as a serious adverse event (SAE) and will be line-listed, as described in “Severe adverse events”.
5. Pulsatility index of umbilical artery: we will use the first pulsatility index measured on ultrasound performed > 24 h after starting study medication.
6. Birthweight (grammes): we will separately describe the birthweight of live-born neonates and the birthweight of fetuses that experienced intra-uterine death.
7. Gestational age of either delivery or intra-uterine death (weeks and days).

### Exploratory outcomes

The relevant exploratory outcomes we plan to report, are listed in Table 2 for mother and fetus/neonate.

**Table 2:** Maternal and fetal/neonatal outcomes

	Intention to treat			Intention to treat, adjusted for GA and EFW at inclusion			Per protocol		
	Sildenafil (*n* =)	Placebo (*n* =)	*P* value	Sildenafil (*n* =)	Placebo (*n* =)	*P* value	Sildenafil (*n* =)	Placebo (*n* =)	*P* value
Maternal outcomes									
Treatment duration (days)									
Gestational age at delivery (weeks + days)									
Pregnancy prolongation after randomisation (days)									
Abdominal circumference at ultrasound closest to 2 weeks after randomisation (mm)									
Mode of delivery									
Caesarean section on fetal indication (%)									
Caesarean section on maternal indication (%)									
Induced vaginal delivery on fetal indication (%)									
Induced vaginal delivery on maternal indication (%)									
Spontaneous vaginal delivery (%)									
Induction of labour after intra-uterine death (%)									
Pregnancy induced hypertension (%)									
Preeclampsia (%)									
HELLP syndrome (%)									
Maternal use of antihypertensive treatment antenatal or postnatal									
One antihypertensive									
Two antihypertensives									
Three or more antihypertensives									
Maternal need for magnesium sulphate for hypertension (%)									
Neonate born between 48 h and 14 days after antenatal corticosteroids course (complete course) (%)									
Neonate born between 0 and 48 h after antenatal corticosteroids course (incomplete course) (%)									
Neonate born during maternal administration of intravenous magnesium sulphate (%)									
Fetal/neonatal outcomes									
Intra-uterine death (%)									
Neonatal death (%)									
Survival at hospital discharge (%)									
Survival with relevant morbidity at hospital discharge (%)									
Survival without relevant morbidity at hospital discharge (%)									
Birth weight of neonates with intra-uterine death (grammes)									
Birth weight of neonates with live birth (grammes)									
Postmenstrual age at first discharge home (weeks + days)									
IVH grade III or IV (%)									
PVL grade II or more (%)									
Moderate or severe BPD (%)									
No BPD (%)									
ROP treated by laser or surgery (%)									
One or more culture-proven episode of infection or clinical episode of infection with antibiotic treatment necessary ≥ 5 days (%)									
NEC grade II or more (%)									

*GA* gestational age, *EFW* estimated fetal weight, *HELLP* haemolysis, elevated liver enzymes, and low platelets syndrome, *IVH* intraventricular hemorrhage, *PVL* periventricular leukomalacia, *BPD* bronchopulmonary dysplasia, *ROP* retinopathy of prematurity, *NEC* necrotising enterocolitis

The percentage of infants that have been assessed for each particular diagnosis will be described for all neonatal outcomes. A table will be presented with line-listing of the primary causes of neonatal death as well. Frequencies and the proportion of total neonatal deaths will be reported.

### Severe adverse events

Severe adverse events (SAEs) were pre-defined as any medical occurrence that results in death, is life-threatening, causes or prolongs hospital admission, results in persistent or significant disability or incapacity, or results in congenital anomaly. Due to the characteristics of the included patient group, mortality, morbidity, and hospital admission are common. In the study protocol maternal and fetal/neonatal SAEs were divided into a group of “context-specific” and “non-context-specific” SAEs. Fetal/neonatal context-specific SAEs consist of the events that are explained by and related to the prematurity and dysmaturity due to fetal growth restriction, for example intra-uterine death, neonatal death due to complications of prematurity/dysmaturity. Non-context-specific SAEs will be considered to be unfavourable events that are not explained by the prematurity/dysmaturity as a result of the fetal growth restriction. Hospital admission for delivery, hypertensive disorders or fetal monitoring will be considered as context-specific maternal SAEs. Other maternal SAEs will be considered to be non-context-specific. All SAEs are evaluated by the Data Monitoring Committee: the context-specific SAEs are monitored during the safety analysis and performed after every 50 patients that completed the study. Non-context-specific SAEs will be sent to and evaluated by the committee right away.

Due to the character and the expected high prevalence of SAEs we did not define SAEs as primary or secondary outcome and will not perform statistical testing on the SAEs, but report them through line-listing.

### Adverse effects

Patients are asked to keep note of the adverse effects they experience during the use of study medication in order to evaluate the percentage of women experiencing adverse effects and evaluate the character of experienced adverse effects.

### Subgroup analysis

Pre-defined subgroup analyses are:

- An abnormal or normal serum level of placental growth factor (PlGF), defined as PlGF < 5th percentile of the reference value and ≥ 5th percentile of the reference value
- Placental growth factor (PlGF) < 25th percentile of all samples of the study population and PlGF ≥ 25th percentile of all samples of the study population
- Gestational age at inclusion, categorized as < 25 weeks of gestation and ≥ 25 weeks of gestation.
- Estimated fetal weight (EFW) at inclusion, categorised as < 300 g, 300–599 g, and ≥ 600 g.
- Neonates that appear to have a congenital anomaly, which was not known in the antenatal period, and thus at the time of randomisation, will be included in the final analysis. However, we propose a subgroup analysis in this group of patients and if we find a significant difference in the primary outcome of these neonates, we will consider excluding them.

We plan to perform a prognostic study and aim to have the methodology published in a separate statistical analysis plan.

### Stratification and design variables

The only stratification variable in the randomisation will be trial site (hospital). 11 Hospitals participated in the study.

### Sample size and power estimations

The sample size of the Dutch STRIDER trial has been previously estimated [59]. With an acceptable risk of type I error of 5% and risk of type II error of 80% we aim to investigate a decrease on the primary outcome from 71% [23] in the control group to 56% in the experimental group, which is equal to a relative risk reduction just above 21%. Allowing for one interim analysis according to the O’Brien-Fleming spending function (*p* < 0.005), 175 women are needed per group. This sensitivity analysis was taken into account in the sample size analysis, if the anticipated inclusion target is reached the final analysis will still powered at 80% to test at a significance level of 0.05. We will include an extra 10 women to account for loss to follow up. The total sample size has been modified to 360 women.

A total of 796 patients will be participating if all STRIDER trials include the number of patients indicated in the sample size calculations. With this number of participants, we will have 80% power to detect a difference of 8.6% in the primary outcome between the intervention and placebo group, having a risk of 5% type I error.

Power estimations for secondary outcomes: based on the estimated sample size of 360 women and an acceptable risk of type I error of 5%, we estimated the statistical power of the secondary outcomes:

1. Neurodevelopmental impairment: 60% power to confirm or reject an increase in neurodevelopmental impairment from 10% [60] in the control group to 20% in the experimental group, equal to a relative risk reduction of just above 21%, having a risk of 5% for type I error.
2. Bayley III score: 80% power to confirm or reject a minimal relevant difference of 5.5 points on the mean composite motor score of the Bayley scales of infant development BSID-III [56], when assuming that 148 children will be alive at 2 years of age and that the mean composite score in the placebo group is 99 (SD 12), with an acceptable risk of 5% for type I error [60].
3. The proportion of mothers experiencing either preeclampsia or HELLP syndrome: 80% power to detect an increase from 50% [23, 26, 61] in the placebo group to 65% in the sildenafil group.
4. Pulsatility index (PI) of the umbilical artery: 80% power to confirm or reject a mean difference of 0.03 in PI, when assuming that PI before sildenafil administration is 1.13 (SD 0.10) [22] with an acceptable risk of 5% for type I error.
5. Birthweight (grammes): 80% power to confirm or reject a mean difference of 45 g in the birthweight, when assuming the mean birthweight in the placebo group is 422 g (SD 159) with an acceptable risk of 5% for type I error [23].
6. Gestational age at either delivery or intra-uterine death: 94% power to confirm or reject a mean difference of one week in the gestational age at delivery (SD 2.7 weeks [26]).

### Interim analysis

Safety analyses are planned after every 50 patients completing the trial (defined as hospital discharge of the neonate) in which no statistical testing will be performed. The Data Safety Monitoring Committee (DSMB) consists of gynecologists and neonatologist and an independent statistician [62]. One interim analysis is planned after outcomes are available for the first half of the anticipated 180 patients have completed the trial. During the interim analysis, the trial will be stopped if a significant difference in primary outcome between the two treatment arms is observed (*p* < 0.005 according to the O’Brian-Fleming rule) [63]. The study can be stopped at any time in case the safety of the patients or the fetus is considered to be in danger. Also, evidence from other trials and data from the ongoing STRIDER trials will be considered during interim analysis [64].

### Statistical analysis

Data on all outcomes will be analysed by two independent statisticians blinded to treatment allocation. Two independent statistical reports will be sent to a third statistician and if there are discrepancies, then the three statistical experts will discuss possible reasons and identify the most correct result.

### General analysis principles

The analysis of the Dutch STRIDER trial will be an intention-to-treat analysis, including all patients randomised in the trial. Random intercept models will be used for all primary analyses to account for a centre effect. This method assumes that the effect is constant across the centres, but that the background risks differ. Additionally, we will secondly also adjust all primary analyses for design variables by adding them to the regression model. The design variables will be estimated fetal weight at inclusion and gestational age at inclusion. The course of pregnancy can be difficult to predict. In some women, there will unexpectedly be signs of fetal distress or worsening of the maternal condition due to a hypertensive disorder and therefore emergency delivery might be necessary, even before starting study medication. Therefore, a per-protocol analysis is planned as well, including only patients that used at least one tablet of study medication.

STATA 15 will be used for the statistical analysis and analysis is planned to follow the 5-step procedure for evaluation of intervention effects in randomised clinical trials, as proposed by Jakobsen et al. [65]. The five steps consist of (1) reporting the confidence intervals and the exact *P* values for the primary, secondary, and exploratory outcomes; (2) reporting Bayes factor for the primary outcome; (3) adjusting the confidence intervals and the statistical significance threshold if the trial is stopped early or if interim analyses have been conducted [66, 67]; (4) adjusting the confidence intervals and the *P* values for multiplicity due to number of outcome comparisons; and (5) assessing clinical significance of the trial results.

We plan to publish the results of the trial in a primary publication, reporting the primary and secondary outcomes assessed at discharge home of the neonate. The results of the 2-year neurodevelopmental assessment will be published separately.

The Bayes factor is the ratio between the probability of obtaining the result assuming the null hypothesis (H_0_) is true divided by the probability of obtaining the result assuming the alternative hypothesis (H_A_) is true. This factor will be calculated, as the *P* value may be misleading in the case of a low probability of the trial results being compatible with the hypothetical intervention effect in the sample size calculation, even though the *P* value is below the pre-specified threshold [68]. A result < 1.0 supports the conclusion that the sildenafil improves healthy survival in fetal growth restriction, while a Bayes factor > 1.0 supports the inverse conclusion. The suggested threshold in the literature is 0.1 for Bayes factor as an indicator of a high probability of an intervention effect similar to or even greater than the hypothetical intervention effect used in the sample size calculation.

Dichotomised outcomes will be presented as proportions of participants in each group with the event, and risk ratios with 95% confidence intervals. Relative risks will be analysed using generalised linear models (bireg) using a log link function [69]. Additionally, absolute risk reductions and number needed to treat will be presented for interpretability.

Continuous outcomes will be presented as means, standard deviations, and 95% confidence intervals or medians and interquartile ranges for each group and mean differences, standard deviations, and 95% confidence intervals for the difference between the groups. Continuous outcomes will be analysed using linear regression.

### Missing data

In the case of missing data, we will follow the principles described by Jakobsen et al. [70] and decide how to handle missing data based on the type of variable or outcome, type of missingness, and proportion of missing data. Either complete case analysis or single or multiple imputation are possible solutions for missing data.

As we expect to have some missing data on the secondary outcome of neurodevelopment, we expect to perform imputation on this outcome. Imputation will not be performed for baseline criteria.

### Outline of figures and tables

**Fig. 1:**
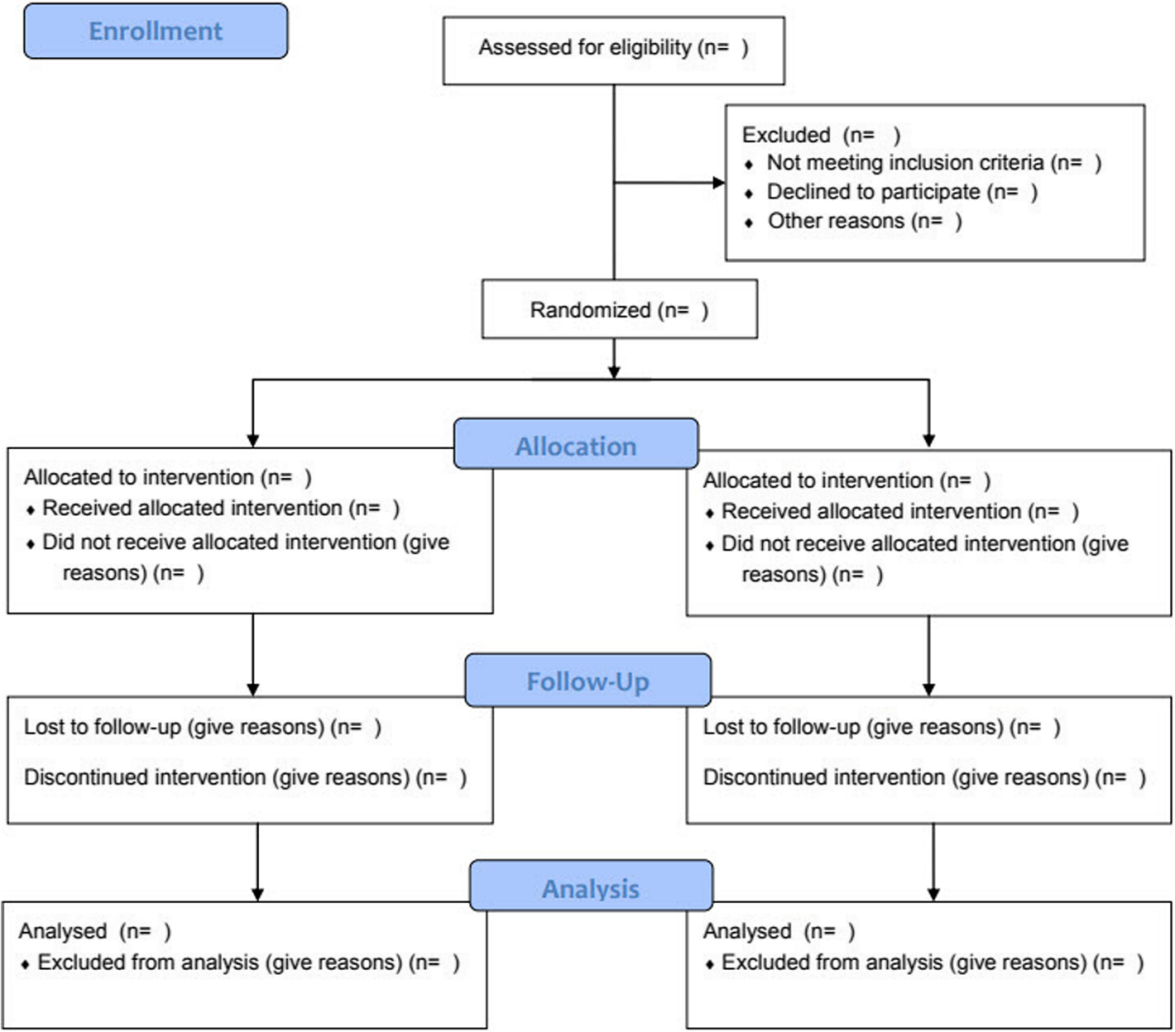
CONSORT 2010 flow diagram

Figure 1 will be the CONSORT diagram with the flow chart of eligible and randomised patients.

Table 1 will be the table with baseline criteria. The maternal and fetal/neonatal outcomes will be expressed in Table 2, showing both the intention-to-treat and the per-protocol analysis. The neonatal outcomes will not be available for all patients, as some patients will have died before assessing a certain variable, for example bronchopulmonary dysplasia, which is assessed at 36 weeks of gestation. In the table will be noted how many neonates have been assessed for that specific variable. A table will be presented with line-listing of the primary causes of neonatal death as well. Frequencies and proportion of total neonatal deaths will be shown.

Table 3 will express the Doppler measurements at inclusion and first measurement after starting medication (at least 24 h after starting medication) will be expressed for treatment allocation and will only show the women who at least had one Doppler measurement after inclusion.

**Table 3:** Doppler measurements at inclusion and first measurement > 24 h after start medication

	Sildenafil (*n* =)		Placebo (*n* =)	
	At inclusion	After starting medication	At inclusion	After starting medication
Mean PI uterine artery				
PI umbilical artery				
PI middle cerebral artery				
PI ductus venosus				

*PI* pulsatility index

Non-context-specific maternal and fetal/neonatal SAE’s in both treatment groups will be line-listed in a table (Table 4) and the maternal side effects of the study medication will be expressed in Table 5 per treatment allocation. Table 6 will express the 2-year neurodevelopmental outcomes and Table 7 the physical outcomes at 2 years. Tables 6 and 7 will not be part of the primary publication, but will be published separately.

**Table 4:** Line-listing of non-context specific SAEs

	Sildenafil (*n* =)	Placebo (*n* =)
Maternal		
…		
…		
Other, namely: …		
Fetal/neonatal		
…		
…		
Other, namely: …		

*SAE* serious adverse event

**Table 5:** Adverse effects of study medication

	Sildenafil (*n* =)	Placebo (*n* =)
Headache (%)		
Flushing (%)		
Stuffy nose (%)		
…		
Other

**Table 6:** Two-year neurodevelopmental outcomes

	Intention to treat			Intention to treat, adjusted for GA and EFW at inclusion			Per protocol		
	Sildenafil (*n* =)	Placebo (*n* =)	*P* value	Sildenafil (*n* =)	Placebo (*n* =)	*P* value	Sildenafil (*n* =)	Placebo (*n* =)	*P* value
Cognitive composite score (mean)									
Motor score (mean)									
Fine motor score (mean)									
Gross motor score (mean)									
Bayley III cognitive composite score and motor score									
< 70									
70–84									
85–99									
≥ 100									
Bayley III motor composite score and motor score									
< 70									
70–84									
85–99									
≥ 100									
Cerebral palsy, all*									
GMFCS grade 1									
GMFCS grade 2									
GMFCS grade 3									
GMFCS grade 4									
GMFCS grade 5									
Normal vision									
Impaired vision despite glasses or lenses									
Mildly abnormal vision despite glasses or lenses									
No useful vision									
Strabismus or amblyopia with normal (corrected) vision									
Normal hearing									
Subnormal hearing for those cases that do need aids and have mild hearing loss at time of testing at age 2 years (i.e. mostly conductive in origin)									
Hearing loss (partly) corrected with aids									
Hearing loss not corrected with aids									
Normal communication									
No normal communication									
Growth									
height mean z-score, corrected age									
Weight mean z-score corrected age BMI z-score corrected age									
Head circumference mean z-score corrected age									
Neurodevelopmental impairment I and II**									

*GA* gestational age, *EFW* estimated fetal weight, *GMFCS* Gross Motor Function Classification System

*We will score all cerebral palsy (CP) cases and then subdivide them in GMFCS levels; a child that does not have CP will not have a GMFCS score

**Defined as either a cognitive Bayley III score < 85 or estimated cognitive delay > 3 months, cerebral palsy, with a GMFCS > 1, hearing loss needing hearing aids, or severe visual loss (legally certifiable as blind or partially sighted)

**Table 7:** Physical outcomes at 2 years

	Intention to treat			Intention to treat, adjusted for GA and EFW at inclusion			Per protocol		
	Sildenafil (*n* =)	Placebo (*n* =)	*P* value	Sildenafil (*n* =)	Placebo (*n* =)	P value	Sildenafil (*n* =)	Placebo (*n* =)	P value
Number of readmissions since primary discharge									
Number of surgery procedures since primary discharge									
Number of medications used in last year									
Current medication use									

### Changes between the protocol and the statistical analysis

The primary outcome in the original protocol is stated as “intact survival at term age”. For the purpose of the analysis we will express the primary outcome as a composite outcome of mortality and survival with major morbidity. In the outcome table the distinction will be made between the proportion of patients that have intra-uterine death and that have neonatal death. Also, survival without major morbidity and the proportions of neonates surviving with the different morbidities including the grades will be reported separately.

Other changes between the original protocol and the proposed statistical analysis presented here are the sample size calculation, as the stopping rule was changed from Haybittle- Peto to the Lan-DeMets-O’Brian Fleming-rule to avoid early stopping of the trial if sildenafil seems to be more effective than placebo [67].

### Patient and public involvement

The development of the research question, outcome measures, and trial design was based on expert consensus in an international collaboration [31]. No patients were involved in the design stage of the randomised controlled trial. However, patient representatives of the relevant patient organizations were consulted for the funding application and they eagerly supported the trial and recommended it for funding. No patients were involved in the recruitment to and conduct of the study. After completion of the study, study participants will be informed by the study team about the results and the drug allocation received. The burden of the intervention was not assessed by patients themselves. The dissemination of the results will also be through the relevant patient organisations.

## Current trial status

At the moment of submission of this manuscript, the number of inclusions was 186, which corresponds to 52% of anticipated sample size. However, during interim analysis performed on 19 July 2018, evaluating the results of the first 183 patients, the DSMB had advised stopping the trial due to safety concerns and a lack of evidence of positive effects. At that time, 216 patients (60% of anticipated sample size) were recruited in in the trial. The patients that were still using study medication stopped taking the tablets. The treatment allocation of all patients was unblinded and was seen by the researchers. This manuscript was submitted on 15 March and was under review.

Despite the smaller sample size and early unblinding of the drug allocation, we will try as much as possible to perform the analyses according to the previously described statistical analysis plan. The consequence is that our study might not have enough power for the primary and all of the secondary outcomes. The performance of the previously planned IPD meta-analysis with the other STRIDER trials will become more important. We plan to analyse patients that stopped taking the study medication due to the stopping of the trial, in both the intention-to-treat and in the per-protocol analyses. However, we will perform subgroup analysis in which we will exclude these patients to see whether this will change the primary and secondary outcomes significantly.

## Discussion

With the described statistical analysis plan we tried to minimise the risks of reporting bias and data-drive analysis in reporting the main results of the Dutch STRIDER trial. We described the pre-defined baseline criteria and primary and secondary outcomes and the analysis plan per outcome.

Four other STRIDER trials with similar inclusion criteria, intervention, and outcome measures are undertaken simultaneously. By performing an individual patient data (IPD) meta-analysis over the results of the five trials, more reliable conclusions can be drawn than from this single trial. However, until all the trials have been performed and individually analysed, we hope that the described statistical approach for the Dutch STRIDER trial will help give a temporary answer to the question of whether or not sildenafil increases the chance of healthy survival in women with severe early-onset fetal growth restriction and whether or not this therapy needs to be applied in clinical practice.

## Conclusions

The Dutch STRIDER trial investigates if sildenafil compared with placebo increases the chance of intact neonatal survival at term age in pregnancies complicated by fetal growth restriction. The present statistical analysis plan for the main outcomes of this trial is presented to minimise the risk of reporting bias and data-driven analysis. The results may have profound effects on the health and quality of life of 700–900 patients in The Netherlands each year, and globally the number could be 700,000 patients.

## Abbreviations

ALT: Alanine aminotransferase
AST: Aspartate aminotransferase
BMI: Body mass index
BPD: Bronchopulmonary dysplasia
BSID: Bayley Scales of Infant Development
CONSORT: Consolidated standards of reporting trials
CP: Cerebral palsy
CRF: Case record form
DSMB: Data safety monitoring board
EFW: Estimated fetal weight
FGR: Fetal growth restriction
GCP: Good clinical practice
GMFCS: Gross Motor Function Classification System
HELLP: Haemolysis, elevated liver enzymes, and low platelets syndrome
IQR: Inter quartile range
IVH: Intraventricular hemorrhage
LDH: Lactate dehydrogenase
Mg: Milligram
MRI: Magnetic resonance imaging
NDI: Neurodevelopmental impairment
NEC: Necrotising enterocolitis
NICHB: National Institute of Child Health and Human Development
PDE-5: Phosphodiesterase-5
PI: Pulsatility index
PlGf: Placental growth factor
PVL: Periventricular leukomalacia
ROP: Retinopathy of prematurity
SAE: Severe adverse event
SD: Standard deviation
SGA: Small for gestational age
STRIDER: Sildenafil therapy in dismal prognosis early-onset fetal growth restriction

## References

[1] Gordijn SJ (2016). Consensus definition of fetal growth restriction: a Delphi procedure. Ultrasound Obstet Gynecol.

[2] Nardozza LM (2017). Fetal growth restriction: current knowledge. Arch Gynecol Obstet.

[3] Severi FM (2000). Intrauterine growth retardation and fetal cardiac function. Fetal Diagn Ther.

[4] Cauli O (2010). Treatment with sildenafil prevents impairment of learning in rats born to pre-eclamptic mothers. Neuroscience.

[5] Herraiz S (2012). Sildenafil citrate improves perinatal outcome in fetuses from pre-eclamptic rats. BJOG.

[6] Karasu E (2012). Endothelial dysfunction in the human umbilical artery due to preeclampsia can be prevented by sildenafil. Clin Exp Hypertens.

[7] Maharaj CH (2009). Effects and mechanisms of action of sildenafil citrate in human chorionic arteries. Reprod Biol Endocrinol.

[8] Miller SL (2009). The effects of sildenafil citrate (Viagra) on uterine blood flow and well being in the intrauterine growth-restricted fetus. Am J Obstet Gynecol.

[9] Nassar AH (2012). Effects of sildenafil in Nomega-nitro-L-arginine methyl ester-induced intrauterine growth restriction in a rat model. Am J Perinatol.

[10] Pellicer B (2011). Haemodynamic effects of long-term administration of sildenafil in normotensive pregnant and non-pregnant rats. BJOG.

[11] Ramesar SV (2011). Sildenafil citrate decreases sFlt-1 and sEng in pregnant l-NAME treated Sprague-Dawley rats. Eur J Obstet Gynecol Reprod Biol.

[12] Samangaya RA (2009). A randomised, double-blinded, placebo-controlled study of the phosphodiesterase type 5 inhibitor sildenafil for the treatment of preeclampsia. Hypertens Pregnancy.

[13] Sanchez-Aparicio P (2008). Effects of sildenafil on the fetal growth of guinea pigs and their ability to survive induced intrapartum asphyxia. Am J Obstet Gynecol.

[14] Sasser JM, Baylis C (2010). Effects of sildenafil on maternal hemodynamics and fetal growth in normal rat pregnancy. Am J Physiol Regul Integr Comp Physiol.

[15] Satterfield MC (2010). Sildenafil citrate treatment enhances amino acid availability in the conceptus and fetal growth in an ovine model of intrauterine growth restriction. J Nutr.

[16] Stanley JL (2012). Sildenafil citrate rescues fetal growth in the catechol-O-methyl transferase knockout mouse model. Hypertension.

[17] Turgut NH (2008). The effect of sildenafil on the altered thoracic aorta smooth muscle responses in rat pre-eclampsia model. Eur J Pharmacol.

[18] Villanueva-Garcia D (2007). A systematic review of experimental and clinical studies of sildenafil citrate for intrauterine growth restriction and pre-term labour. J Obstet Gynaecol.

[19] Wareing M (2005). Sildenafil citrate (Viagra) enhances vasodilatation in fetal growth restriction. J Clin Endocrinol Metab.

[20] Wareing M (2006). Phosphodiesterase-5 inhibitors and omental and placental small artery function in normal pregnancy and pre-eclampsia. Eur J Obstet Gynecol Reprod Biol.

[21] Chen J (2016). Effect of L-arginine and sildenafil citrate on intrauterine growth restriction fetuses: a meta-analysis. BMC Pregnancy Childbirth.

[22] Dastjerdi MV, Hosseini S, Bayani L (2012). Sildenafil citrate and uteroplacental perfusion in fetal growth restriction. J Res Med Sci.

[23] von Dadelszen P (2011). Sildenafil citrate therapy for severe early-onset intrauterine growth restriction. BJOG.

[24] Trapani A (2016). Comparison between transdermal nitroglycerin and sildenafil citrate in intrauterine growth restriction: effects on uterine, umbilical and fetal middle cerebral artery pulsatility indices. Ultrasound Obstet Gynecol.

[25] Gluud C (2007). Hepatology may have problems with putative surrogate outcome measures. J Hepatol.

[26] Trapani A (2016). Perinatal and hemodynamic evaluation of sildenafil citrate for preeclampsia treatment: a randomized controlled trial. Obstet Gynecol.

[27] Hrobjartsson A (2014). Bias due to lack of patient blinding in clinical trials. A systematic review of trials randomizing patients to blind and nonblind sub-studies. Int J Epidemiol.

[28] Savovic J (2012). Influence of reported study design characteristics on intervention effect estimates from randomized, controlled trials. Ann Intern Med.

[29] Schulz KF (1995). Empirical evidence of bias. Dimensions of methodological quality associated with estimates of treatment effects in controlled trials. JAMA.

[30] Sutton AJ (2000). Empirical assessment of effect of publication bias on meta-analyses. BMJ.

[31] Ganzevoort W (2014). STRIDER: sildenafil therapy in dismal prognosis early-onset intrauterine growth restriction--a protocol for a systematic review with individual participant data and aggregate data meta-analysis and trial sequential analysis. Syst Rev.

[32] Andrew Sharp CC, Jackson R, Harrold J, Turner MA, Kenny LC, Baker PN, Johnstone ED, Khalil A, von Dadelszon P, Papageorghiou AT, Alfirevic Z, on behalf of the STRIDER group. Maternal sildenafil for severe fetal growth restriction (STRIDER): a multicentre, randomised, placebo-controlled, double-blind trial. Lancet Child Adolesc Health Published Online. 2017. Lancet Child Adolesc Health. 2018;2(2):93-102. 10.1016/S2352-4642(17)30173-6 30169244

[33] Ganzevoort W (2014). Dutch STRIDER study protocol.

[34] Boutron I (2007). Reporting methods of blinding in randomized trials assessing nonpharmacological treatments. PLoS Med.

[35] Hrobjartsson A (2012). Observer bias in randomised clinical trials with binary outcomes: systematic review of trials with both blinded and non-blinded outcome assessors. BMJ.

[36] Hrobjartsson A (2013). Observer bias in randomized clinical trials with measurement scale outcomes: a systematic review of trials with both blinded and nonblinded assessors. CMAJ.

[37] Hrobjartsson A (2014). Observer bias in randomized clinical trials with time-to-event outcomes: systematic review of trials with both blinded and non-blinded outcome assessors. Int J Epidemiol.

[38] Page MJ (2016). Empirical evidence of study design biases in randomized trials: systematic review of meta-epidemiological studies. PLoS One.

[39] Savovic J (2012). Influence of reported study design characteristics on intervention effect estimates from randomised controlled trials: combined analysis of meta-epidemiological studies. Health Technol Assess.

[40] Wood L (2008). Empirical evidence of bias in treatment effect estimates in controlled trials with different interventions and outcomes: meta-epidemiological study. BMJ.

[41] WMA Declaration of Helsinki – ethical principles for medical research involving human subjects. 2013. [Available from: https://www.wma.net/policies-post/wma-declaration-of-helsinki-ethical-principles-for-medical-research-involving-human-subjects/. 19886379

[42] Wet medisch-wetenschappelijk onderzoek met mensen. 2017; Available from: http://wetten.overheid.nl/BWBR0009408/2017-03-01.

[43] Medical Research Involving Human Subjects (Medical Research (Human Subjects) Act. Available from: https://english.ccmo.nl/investigators/legal-framework-for-medical-scientific-research.

[44] De experimentenwet (7 mei 2004) en Uitvoeringsbesluit KB (koninklijk besluit) (30 juni 2004). Available from: https://www.fagg-afmps.be/en/human_use/medicines/medicines/research_development/clinical_trials.

[45] Guideline for Good Clinical Practice. Available from: http://www.ich.org/fileadmin/Public_Web_Site/ICH_Products/Guidelines/Efficacy/E6/E6_R1_Guideline.pdf.

[46] Kessler KM (2002). The CONSORT statement: explanation and elaboration. Consolidated Standards of Reporting Trials. Ann Intern Med.

[47] L.M. Leijser, G.M., S.M. Mulder-de Tollenaer, Aanbeveling neonatale neuroimaging versie 1.5. 2015.

[48] Bancalari E, Claure N (2006). Definitions and diagnostic criteria for bronchopulmonary dysplasia. Semin Perinatol.

[49] Finer NN, Bates R, Tomat P (1996). Low flow oxygen delivery via nasal cannula to neonates. Pediatr Pulmonol.

[50] Jobe AH, Bancalari E (2001). Bronchopulmonary dysplasia. Am J Respir Crit Care Med.

[51] Walsh MC (2003). Safety, reliability, and validity of a physiologic definition of bronchopulmonary dysplasia. J Perinatol.

[52] Walsh MC (2004). Impact of a physiologic definition on bronchopulmonary dysplasia rates. Pediatrics.

[53] Kindergeneeskunde, N.V.v (2013). Richtlijn Bronchopulmonaire dysplasie.

[54] (NOG), N.O.G (2012). Richtlijn Prematuren Retinopathie.

[55] Bell MJ (1978). Neonatal necrotizing enterocolitis. Therapeutic decisions based upon clinical staging. Ann Surg.

[56] Bayley N (2006). Bayley Scales of Infant and Toddler Development—Third Edition: Administration manual.

[57] (NVOG), N.V.v.O.e.G. (2011). Hypertensieve aandoeningen in de zwangerschap.

[58] Saphier CJ, Repke JT (1998). Hemolysis, elevated liver enzymes, and low platelets (HELLP) syndrome: a review of diagnosis and management. Semin Perinatol.

[59] Onderzoeksprotocol The Dutch STRIDER 10.5281/zenodo.56148.

[60] Lees CC (2015). Two-year neurodevelopmental and intermediate perinatal outcomes in infants with very preterm fetal growth restriction (TRUFFLE): a randomised trial. Lancet.

[61] Lees C (2013). Perinatal morbidity and mortality in early-onset fetal growth restriction: cohort outcomes of the trial of randomized umbilical and fetal flow in Europe (TRUFFLE). Ultrasound Obstet Gynecol.

[62] Dutch STRIDER: Data Monitoring Committee Charter 10.5281/zenodo.56147.

[63] O'Brien PC, Fleming TR (1979). A multiple testing procedure for clinical trials. Biometrics.

[64] Ashby D, Machin D (1993). Stopping rules, interim analyses and data monitoring committees. Br J Cancer.

[65] Jakobsen JC (2014). The thresholds for statistical and clinical significance - a five-step procedure for evaluation of intervention effects in randomised clinical trials. BMC Med Res Methodol.

[66] Wetterslev J, Jakobsen JC, Gluud C (2017). Trial sequential analysis in systematic reviews with meta-analysis. BMC Med Res Methodol.

[67] Jakobsen, J.C., Systematic reviews of randomised clinical trials examining the effects of psychotherapeutic interventions versus ‘no intervention’ for major depressive disorder and a randomised trial examining the effects of ‘third wave’ cognitive therapy versus mentalization-based treatment for major depressive dis-order. Ph.D. Thesis, 2013: p. 1–62. 25283628

[68] Goodman SN (2005). Introduction to Bayesian methods I: measuring the strength of evidence. Clin Trials.

[69] McNutt LA (2003). Estimating the relative risk in cohort studies and clinical trials of common outcomes. Am J Epidemiol.

[70] Jakobsen JC (2017). When and how should multiple imputation be used for handling missing data in randomised clinical trials - a practical guide with flowcharts. BMC Med Res Methodol.

